# *ARID5B* polymorphism confers an increased risk to acquire specific *MLL* rearrangements in early childhood leukemia

**DOI:** 10.1186/1471-2407-14-127

**Published:** 2014-02-25

**Authors:** Mariana Emerenciano, Thayana Conceição Barbosa, Bruno Almeida Lopes, Caroline Barbieri Blunck, Alessandra Faro, Camilla Andrade, Claus Meyer, Rolf Marschalek, Maria S Pombo-de-Oliveira

**Affiliations:** 1 Pediatric Hematology-Oncology Program, Research Center, Instituto Nacional de Câncer, Rua André Cavalcanti 37, Rio de Janeiro/RJ 20231-050, Brasil; 2 Institute of Pharmaceutical Biology/ZAFES/Diagnostic Center of Acute Leukemia (DCAL), Goethe-University of Frankfurt, Frankfurt/Main, Germany

**Keywords:** *IKZF1*, *ARID5B*, *CEBPE*, Infant leukemia, *MLL*

## Abstract

**Background:**

Acute leukemia in early age (EAL) is characterized by acquired genetic alterations such as *MLL* rearrangements (*MLL*-r). The aim of this case-controlled study was to investigate whether single nucleotide polymorphisms (SNPs) of *IKZF1*, *ARID5B*, and *CEBPE* could be related to the onset of EAL cases (<24 months-old at diagnosis).

**Methods:**

The SNPs (*IKZF1* rs11978267, *ARID5B* rs10821936 and rs10994982, *CEBPE* rs2239633) were genotyped in 265 cases [169 acute lymphoblastic leukemia (ALL) and 96 acute myeloid leukaemia (AML)] and 505 controls by Taqman allelic discrimination assay. Logistic regression was used to evaluate the association between SNPs of cases and controls, adjusted on skin color and/or age. The risk was determined by calculating odds ratios (ORs) with 95% confidence interval (CI).

**Results:**

Children with the *IKZF1* SNP had an increased risk of developing *MLL*-germline ALL in white children. The heterozygous/mutant genotype in *ARID5B* rs10994982 significantly increased the risk for *MLL*-germline leukemia in white and non-white children (OR 2.60, 95% CI: 1.09-6.18 and OR 3.55, 95% CI: 1.57-8.68, respectively). The heterozygous genotype in *ARID5B* rs10821936 increased the risk for *MLL*-r leukemia in both white and non-white (OR 2.06, 95% CI: 1.12-3.79 and OR 2.36, 95% CI: 1.09-5.10, respectively). Furthermore, *ARID5B* rs10821936 conferred increased risk for *MLL*-*MLLT3* positive cases (OR 7.10, 95% CI:1.54-32.68). Our data do not show evidence that *CEBPE* rs2239633 confers increased genetic susceptibility to EAL.

**Conclusions:**

*IKZF1* and *CEBPE* variants seem to play a minor role in genetic susceptibility to EAL, while *ARID5B* rs10821936 increased the risk of *MLL-MLLT3*. This result shows that genetic susceptibility could be associated with the differences regarding *MLL* breakpoints and partner genes.

## Background

Acute leukemia (AL) is one of the most common malignancies of early childhood. Leukemias in infants (≤12 months) (IL), even being rare, are recurrently studied because they are associated with a high frequency of early death during the first months of life. Despite advances in most other age groups, the prognosis of infants remains poor [1,2]. Therefore, understanding the contributing factors that lead to the emergence of early age leukemia (EAL) represents a major opportunity of prevention. Contributing events include chance, exposure to genotoxic substances, and inherited genetic susceptibility.

Epidemiological and molecular studies have already demonstrated that critical molecular lesions, such as the frequently observed *MLL* gene rearrangements (*MLL*-r) in IL, occur *in utero* in early hematopoietic precursors [3,4]. Maternal exposures during pregnancy seem to be associated with the onset of EALs [5-7]. Many attempts to identify inherited susceptibility in childhood leukemia (as a whole) have been made [8] and some studies have already focused on EAL [9-13]. Common allelic variants in *IKZF1* (7p12.2), *ARID5B* (10q21.2), and *CEBPE* (14q11.2), which are directly related to hematopoietic differentiation and development, have been repeatedly and significantly associated with childhood acute lymphoblastic leukemia (ALL). Of interest, Xu *et al*. presented convincing evidence for modifying effects of genetic *ARID5B* variants; in particular these data consistently show a trend for increasing allelic odds ratio as age decreased and the risk varied substantially by ethnicity [14]. We have made similar observations with age-dependent susceptibility and leukemia emergence EAL [13]. However, the extent to which germline variations contribute to the acquisition of somatic aberrations that define AL subtypes is yet unknown.

Therefore, we genotyped common variants in *IKZF1*, *ARID5B*, and *CEBPE* in a series of children enrolled in the Brazilian Collaborative Study Group of Infant Acute Leukemia (BCSGIAL) in order to evaluate the frequencies of these inherited polymorphisms and determine their associations by (i) age strata (infants *versus* children aged between 13 and 24 months); (ii) *MLL* status and/or type of *MLL*-r; and (iii) ethnic background. From our data we conclude that distinct *ARID5B* rs10821936 polymorphism represents a novel risk factor to the acquisition of somatic mutation as it increases the risk to acquired *MLL*-r in EAL.

## Methods

### Subjects

This study includes samples from 770 Brazilian children (169 ALL, 96 AML and 505 controls) that were ascertained from January, 2003 to December, 2012. They were selected from the BCSGIAL, in which biological material were available. BCSGIAL is a multicentric study, which focuses on investigating the pathogenic mechanisms of EAL in Brazil. Its characteristics and investigations have been published elsewhere [6,15]. Briefly, it consists in a hospital-based case–control study that aims to explore the different risk factors associated with EAL. Cases have been recruited from 15 institutions located throughout all states of the country, but the Amazon. The studied sample of enrolled participants included a ratio of 2 controls per each EAL recruited case in each participating center [6,11,13,15].

Cases and controls were age-matched and from the same Brazilian regions. The exclusion criteria were children with Down syndrome, myelodysplastic syndrome, Fanconi anemia, Bloom’s syndrome, ataxia telangiectasia, neurofibromatosis, and samples with bad quality DNA.

### Leukemia diagnosis

The diagnosis was first established through morphological and immunophenotypic examinations of lymphoid and myeloid cells according to standard criteria. Detection of an *MLL-*r was performed by conventional cytogenetics, reverse transcriptase polymerase chain reaction (PCR), and/or by fluorescence *in situ* hybridisation (LSI *MLL* Dual Color Break Apart Rearrangement Probe, Vysis Inc., IL, USA) as previously described [15]. Long distance inverse PCR (LDI-PCR) was used to identify the *MLL* translocation partner gene (TPG) and the respective breakpoints. Briefly, 1 μg of genomic DNA was digested and the resulting DNA fragments were self-ligated. This re-ligated DNA was used for the subsequent LDI-PCR analysis. PCR amplimers were purified from the gel and subsequently sequenced to obtain chromosomal breakpoint information [16].

### Ethics

Data collection and laboratory procedures were evaluated and approved by the Ethics Committee of all participating hospitals. Data analysis was approved by the Comitê de Ética em Pesquisa (CEP) -Instituto Nacional de Câncer e Comitê Nacional de Ética em Pesquisa (CONEP) (CEP #005/06 and #024/10; CONEP # 707/2010). A written informed consent was obtained from the mothers of the study subjects.

### Genotyping

Genomic DNA was isolated from peripheral blood cells or from buccal cells with the QIAamp DNA Blood Mini Kit (Qiagen, USA) or with Oragene DNA technology (Genotek, Ontario, Canada), respectively, and according to the manufacturer’s instructions. For cases, remission samples were used to isolate genomic DNA. Genotyping of *IKZF1* rs11978267, *ARID5B* rs10821936, *ARID5B* rs10994982, and *CEBPE* rs2239633 was conducted by Taqman allelic discrimination assay (Applied Biosystems: Taqman SNP assays C_199413_10, C_26140184_10, C_30824850_10, and C_335486_1). Genotype calls were made upon visualization of allelic discrimination charts in which the clusters were identified by comparison with reference controls for each allele. To ensure quality of genotyping, 10% of samples were analyzed randomly in duplicates and concordance was absolute.

### Statistical analysis

The expected gene polymorphism frequency was calculated using the Hardy-Weinberg law based on the allele frequency in the control group. To compare the distribution of genotypes between cases and controls the *χ*^2^-test (two-sided) was used (or Fisher’s Exact Test when expected values were less than five). *P*-values ≤0.05 were considered statistically significant. The disease risk associated with SNPs occurrence across overall or subgroups of patients was determined by calculating odds ratios (ORs) with 95% confidence interval (CI). A multivariable logistic regression model (method enter) was used to analyze associations between *ARID5B* variant genotype and subtypes of *MLL*-r [i.e. genomic breakpoint and TPG]. All statistical analyses were performed using the Statistical Product and Services Solutions statistical package, version 18.0 (SPSS Inc, Chicago, IL, USA).

## Results

The call rate for *IKZF1*, *ARID5B* rs10821936 and rs10994982, and *CEBPE* was respectively 247 of 265 (93.2%), 244 of 265 (92.1%), 246 of 265 (92.8%), and 251 of 265 (94.7%) in the investigated cases. The call rate for each SNP was ≥94% in the control groups. Control genotypes for all four SNPs loci were in Hardy-Weinberg equilibrium (*P* > 0.05).

The demographic characteristics of cases and controls are shown in Additional file 1: Table S1. There were no statistical differences among cases and controls regarding gender, ethnicity or children age range. The *MLL* status was established for 149 ALL and 86 AML patients. The analysis of genomic breakpoints by LDI-PCR within the *MLL* breakpoint cluster region was performed in a subset of 55 *MLL*-r with available biological material and successfully determined in 41 cases.

The distribution of allele frequencies among controls and cases within the major acute leukemia subtypes has been evaluated and the results are shown in Additional file 2: Table S2. The risk of developing the pro-B ALL phenotype was increased for patients with the variant allele of *ARID5B* rs10821936 (OR 2.54, 95% CI: 1.36-4.70). Increased risks of developing c-ALL (CD10 positive) have been observed for patients with variant alleles of *ARID5B* rs10821936 (OR 2.63, 95% CI: 1.41-4.90) and rs10994982 (OR 3.13, 95% CI: 1.24-7.95). Among patients with AML, an increased risk has been observed for those patients with the homozygous variant of *ARID5B* rs10821936 (OR 2.39, 95% CI: 1.10-5.17).

The distributions of allele frequencies in controls and cases and the risk association between genetic variants and acute leukemia further stratified by skin color and by *MLL* status are displayed in Additional file 3: Table S3. In overall cases, white and non-white children presented similar risk associations. The heterozygous genotype in *ARID5B* rs10821936 increased the risk for *MLL*-r leukemia in both white and non-white (OR 2.06, 95% CI: 1.12-3.79 and OR 2.36, 95% CI: 1.09-5.10, respectively). The mutant genotype in *ARID5B* SNP rs10821936 significantly increased the risk for *MLL*-germline leukemia in white and non-white children (OR 2.69, 95% CI: 1.28-5.66 and OR 3.69, 95% CI: 1.57-8.68, respectively). The heterozygous/mutant genotype in the other *ARID5B* rs10994982 also significantly increased the risk for *MLL*-germline leukemia in white and non-white children (OR 2.60, 95% CI: 1.09-6.18 and OR 3.55, 95% CI: 1.57-8.68, respectively).

When comparing the ALL cases by age strata (infants *versus* children aged between 13 and 24 months), white children with ALL of both age groups presented with an increased risk for *MLL*-germline leukemia associated with the heterozygous/mutant genotypes *IKZF1* (OR 5.57, 95% CI: 1.39-22.24 and OR 2.58, 95% CI: 1.02-6.51, respectively). The heterozygous genotype in *ARID5B* rs10821936 increased the risk for *MLL*-r ALL in both white and non-white infants (OR 2.19, 95% CI: 1.07-4.49 and OR 3.82, 95% CI: 1.21-12.12, respectively), while for children aged between 13–24 months the mutant genotype significantly increased the risk for ALL in white children, regardless the *MLL* status (OR 7.11, 95% CI: 2.07-24.45 for *MLL*-germline; OR 7.91, 95% CI: 1.47-42.46 for *MLL*-r) (Additional file 3: Table S3).

In AML, the only increased risk association was observed among non-white *MLL*-r cases with the *ARID5B* rs10821936 mutant genotype (OR 4.82, 95% CI: 1.50-15.50), while the *CEBPE* variant allele was negatively associated with *MLL-*germline AML (OR 0.22, 95% CI: 0.07-0.72) (Additional file 3: Table S3).

The SNPs risk associations between acute leukemia and *MLL* status are also shown after statistical adjustment on age and on skin color (Additional file 4: Table S4). The results corroborate with those obtained after stratification, showing that *IKZF1* and *ARID5B* rs10994982 variant alleles play a role in the susceptibility to *MLL*-germline leukemia while *ARID5B* rs10821936 confers increased risk to both *MLL*-germline and *MLL*-r leukemia.

Because the variant *ARID5B* rs10821936 allele was remarkably associated with an increased risk of *MLL*-r acute leukemia, we tested whether this risk allele was associated to a specific *MLL* TPG or to any of the frequent *MLL* breakpoint regions. The risk association between *ARID5B* rs10821936 and *MLL*-r acute leukemia according to the TPGs and *MLL* breakpoint regions compared with controls is shown in Table 1. The individuals with heterozygous/mutant genotype had a higher risk of developing *MLL-AFF1* positive leukemia (OR 2.79, 95% CI: 1.27-6.11) and even higher odds of *MLL-MLLT3* positive leukemia (OR 7.10, 95% CI: 1.54-32.68). Moreover, this increased risk magnitude was also observed for individuals with *MLL* breakpoints non-located in *MLL* intron 11 (OR 10.25, 95% CI: 2.24-46.81). A multivariate analysis has been performed to address whether the *MLLT3* TPG and the *MLL* breakpoint region (exon 9-intron 10) were variables dependent on each other. The results showed that the susceptibility risk of having the *MLL* breakpoint localized outside of *MLL* intron 11 [(OR 0.88, 95% CI: 0.34–2.30), *P* = 0.79] and the *MLLT3* as the TPG [(OR 1.49, 95% CI: 0.86–2.58), *P* = 0.15] is cross-dependent.

**Table 1:** **The risk associations between *ARID5B *** **rs10821936 genotype and *MLL *** **translocation partner genes or *MLL *** **breakpoint region, Brazil, 2003-2013**

	**Controls**	***MLL *** **translocation partner genes**						***MLL *** **breakpoint region**^**a**^			
			***MLL-AFF1 *** **(n = 44)**		***MLL-MLLT1 *** **(n = 21)**		***MLL-MLLT3 *** **(n = 17)**		**A (n = 15) and B (n = 8)**		**C (n = 18)**
	**n**	**n**	**OR (95% CI)**^**b,c**^	**n**	**OR (95% CI)**^**b,c**^	**n**	**OR (95% CI)**^**b,c**^	**n**	**OR (95% CI)**^**b,c**^	**n**	**OR (95% CI)**^**b,c**^
*ARID5B*											
rs10821936											
TT	200	11	1.00	6	1.00	2	1.00	2	1.00	5	1.00
TC	205	27	**3.26 (1.42-7.49)**	11	2.82 (0.89-8.93)	12	**8.62 (1.77-41.94)**	17	**12.76 (2.66-61.23)**	12	**3.45 (1.08-11.05)**
CC	68	6	1.95 (0.62-6.11)	4	2.12 (0.52-8.63)	3	5.18 (0.79-34.02)	4	**6.65 (1.08-41.15)**	1	0.85 (0.09-8.25)
TC + CC	273	33	**2.79 (1.27-6.11)**	15	2.53 (0.89-7.22)	15	**7.10 (1.54-32.68)**	22	**10.25 (2.24-46.81)**	14	2.72 (0.88-8.43)

n, number of individuals; OR, odds ratio; CI, confidence intervals; ^a^The *MLL* breakpoint region was subdivided according to Meyer et al., 2013^32^ as follows: (A) Exon 9-Intron 9, (B) Exon 10-Intron 10, (C) Intron 11; ^b^Adjusted on age; ^c^Adjusted on skin color.

We further tested the effect of cumulative variant alleles of *IKZF1*, *ARID5B* and *CEBPE* in the risk susceptibility to EAL (Additional file 5: Table S5). Patients harboring 6–8 variant alleles had significant increased risk to develop ALL older than 12 months-old (OR 1.34, 95% CI: 1.09-1.66) or *MLL*-germline leukemia (OR 1.33, 95% CI: 1.06-1.67). However, we could not observe a trend for increasing ORs as the number of risk alleles increased.

## Discussion

The molecular epidemiological approach in several genetic studies has raised the concept that most, if not all, childhood leukemia cases originate *in utero*[4]. Previous evidences suggested that the causality factors are likely to be multiple and leukemia subtype-specific, combining both genetic susceptibility and environmental exposures [17]. Moreover, whether and how the inherited gene variants contribute to the acquisition of the *in utero*-acquired somatic alterations frequently found in EAL must be explored.

In this case–control study, we genotyped known susceptibility loci (*IKZF1*, *ARID5B*, and *CEBPE*) in a series of children enrolled in the BCSGIAL. We observed an increased magnitude of ALL risk for children with SNPs in *IKZF1* and *ARID5B*. This is expected from the previous genome wide association studies (GWAS) that have been performed in childhood ALL (peak incidence 2–5 years-old) [18,19]. Our data do not show evidence that *CEBPE* rs2239633 confers increased genetic susceptibility to EAL, in agreement with previous data in IL [12]. In a recent GWAS, *CEBPE* SNPs were strongly related to ALL risk in European Americans, with variable effects in non-European populations [14]. This result could explain the lack of association in our population.

*IKZF1* rs11978267 was associated with the increased risk of *MLL*-germline ALL in both infants and older children consistent with results found in previous settings of childhood ALL. Different from ours, the only previous study that has also addressed involvement of *IKZF1* polymorphism in AML has found a contribution of rs11978267 to susceptibility in infant AML overall, irrespective of *MLL*-r [12]. However, because of the differences in number of cases and ethnicity among studied populations, it is difficult to draw conclusions from this comparison. Therefore, further studies focusing on AML will be necessary to verify the *IKZF1* susceptibility role in EAL. As this is an extremely rare disease, pooling studies would be of great interest.

*ARID5B* gene variants have been systematically shown to increase the risk of childhood ALL in various populations [14,18-23]. Most of these studies showed that this risk was associated to B-cell precursor ALL, and some of them could distinguish B-hyperdiploid ALL from other subtypes [18,19,24]. This association with B-hyperdiploid ALL has not been reproduced in all studies [25]. Overall, the *ARID5B* gene variants were strongly associated with the risk of EAL in this Brazilian series. This gene encodes a member of the AT-rich interaction domain (ARID) family of DNA binding proteins. The encoded protein forms a histone H3K9_me2_ demethylase complex together with PHD finger protein 2 to regulate the transcription of target genes involved in adipogenesis and liver development [26]. An increased risk of *ARID5B* variants in AML had not been reported previously. The gene expression level of *ARID5B* is up-regulated in two different AML subtypes (acute megakaryoblastic and promyelocytic leukemia) [27,28]. Acute megakaryoblastic leukemia is more frequent in EAL AML opposite to promyelocytic leukemia [29,30]. Therefore, it is conceivable that *ARID5B* contributes to susceptibly to EAL AML, and an ongoing case–control study is currently underway to answer this question [31].

The *ARID5B* rs10994982 has only significantly increased the risk in *MLL* germline children, in agreement with observations in childhood [18,19] and IL [12]. We observed a major and wider spectrum of risk increase for *ARID5B* rs10821936. This is consistent with previously mentioned studies, as this specific SNP has been strongly associated with risk across several populations and leukemia subgroups. In our study, the rs10821936 increased the risk for both *MLL* wild-type and *MLL*-r ALL and *MLL*-r AML patients. One of the most significant findings from this study is that *ARID5B* rs10821936 not only differed between EAL and control groups but also distinguished *MLL-MLLT3* positive leukemias from other *MLL*-r. Interestingly, a strong association could be observed both by analyzing the TPG (*MLLT3*) and the breakpoint location of *MLL* (mainly intron 9), and the multivariate model confirmed that these parameters were dependent on each other. Recently, the *MLL* recombinome analysis pointed out different tendencies concerning the breakpoints localization when it was analyzed breakpoint distributions together with TPGs [32]. For that study, the *MLL* breakpoint cluster region was subdivided into 3 sub regions (A, exon 9 - intron 9; B, exon 10 - intron 10; C, exon 11 - intron 12). The observed ‘mean breakpoint frequencies’ for these 3 regions in South America (dataset includes our Brazilian samples) was A = 31.9%, B = 21.7%, and C = 43.5%. However, when separating by *MLLT3* TPG and restricted to the infants subgroup, the MBPF was A = 41.8%, B = 13.3%, and C = 42.9%, while in pediatric and adults these ‘mean breakpoint frequencies’ were: 35.7%, 18.8%, 43.8% and 34.2%, 7.59%, 57.0%, respectively. Therefore, recombination affecting *MLLT3* displayed a tendency for *MLL* intron 9 breaks in IL. Together, all these data are concordant with our finding that increased risk susceptibility in infants is associated with *MLL-MLLT3* rearrangement. Although future studies will be necessary to confirm this finding and to understand the specific role of this SNP in the pathogenesis, the availability of such rare epidemiological set of cases prompted us to suggest an association between inherited gene variants and specific somatic aberrations in the pathogenesis of *MLL*-r EAL.

There are limitations in this present analysis. First, the small number of cases after some subsets stratification raises concern with regards to statistical power. However, given the rarity of this disease, one should consider that the consistency of the associations observed, and the concordance with previously published data indicate good validity and sensitivity of our study. Second, we had missing genotyping calls in some cases and controls that precluded us to have all samples screened uniformly. However, an acceptable call rate has been achieved in either cases or controls and the frequencies obtained did not present any deviation.

We can also mention some study strengths. As replication of GWAS is highly desirable, this is an important contribution of the present Brazilian work, especially because the studies have been so far concentrated to European and American populations. For example, validation sequencing of this *ARID5B* genomic region has been requested in order to reveal the exact nature of the differences previously observed. Moreover, this report focus on EAL and particularly those harboring *MLL*-r, and in this context, this study is innovative.

## Conclusions

In summary, we have shown that *IKZF1* and *CEBPE* studied genetic variants seem to play a minor role in susceptibility to EAL, while *ARID5B* seems to contribute to the multifactorial causes of this disease, even increasing the risk of specific acquired somatic abnormalities such as the *MLL*-r recurrently seen in IL. This knowledge sheds new light onto the complex interactions that exist between environmental factors, inherited polymorphisms, and somatic alterations in leukemogenesis. While improvements on successful therapy have being hard to achieve within very young children, prevention is a major need and, therefore, the clarification of etiology should be tirelessly pursued.

## Competing interests

The authors declare that they have no competing interests.

## Authors’ contributions

ME wrote the manuscript. ME and TCB performed genotyping assays. BAL contributed significantly with controls’ data collection and DNA samples preparation. CBB and CA performed cytogenetic-molecular studies to characterize *MLL* rearrangements. AF performed infant leukaemia cases diagnosis and collected their clinical-demographic data. CM has performed molecular analysis to determine the *MLL* breakpoints. RM provided the conditions to *MLL* breakpoint analysis and contributed with revision of the manuscript. ME and MSPO contributed to the conception of the study, writings and critical analysis of the data. All co-authors of the Brazilian Collaborative Study Group of Infant Acute Leukaemia contributed with clinical and demographical data. All authors read and approved the final manuscript.

## Pre-publication history

The pre-publication history for this paper can be accessed here:

http://www.biomedcentral.com/1471-2407/14/127/prepub

## Supplementary Material

Additional file 1: Table S1 Demographic and biological characteristics of controls and cases according to age at diagnosis. Click here for file

Additional file 2: Table S2 The distribution of allele frequencies among controls and cases within the major acute leukemia subtypes, Brazil, 2003-2013. Click here for file

Additional file 3: Table S3 The distribution of allele frequencies among controls and cases within the two major acute leukemia subtypes by skin colour and by *MLL* gene status, Brazil, 2003-2013. Click here for file

Additional file 4: Table S4. The risk associations between genetic variants and *MLL* status in overall and specific subtypes of acute leukemia, Brazil, 2003-2013. Click here for file

Additional file 5: Table S5 Cumulative risk effects of *IKZF1*, *ARID5B* and *CEBPE* genetic variants according to leukemia subtype and *MLL* status, Brazil, 2003-2013. Click here for file
