# Supplementary material for: ARID5B polymorphism confers an increased risk to acquire specific MLL rearrangements in early childhood leukemia
Source: BMC Cancer. 2014 Feb 25;14:127. doi: 10.1186/1471-2407-14-127 (PMC3948138; doi:10.1186/1471-2407-14-127)
Supplement: Additional file 1: Table S1 — Demographic and biological characteristics of controls and cases according to age at diagnosis. [file 1471-2407-14-127-S1.doc]

**Additional file 1: Table S1:** Demographic and biological characteristics of controls and cases according to age at diagnosis

|  | Controls |  | Cases | | |  | P^c^ |
| --- | --- | --- | --- | --- | --- | --- | --- |
|  | n = 505 |  | All cases n = 265 | ≤12 months n = 148 | 13-24 monthsn = 117 |  |  |
| Gender |  |  |  |  |  |  |  |
| Male | 262 (51.9) |  | 140 (52.8) | 82 (55.4) | 58 (49.6) |  | 0.80 |
| Female | 243 (48.1) |  | 125 (47.2) | 66 (44.6) | 59 (50.4) |  |  |
| Race/skin color^a^ |  |  |  |  |  |  |  |
| White | 303 (60.0) |  | 170 (64.2) | 98 (66.2) | 72 (61.5) |  | 0.26 |
| Non-White | 202 (40.0) |  | 95 (35.8) | 50 (33.8) | 45 (38.5) |  |  |
| Region of Brazil^b^ |  |  |  |  |  |  |  |
| South | 10 (2.0) |  | 29 (10.9) | 19 (12.8) | 10 (8.5) |  | <0.01 |
| Southeast | 353 (69.9) |  | 103 (38.9) | 61 (41.2) | 42 (35.9) |  |  |
| Northeast | 111 (22.0) |  | 87 (32.8) | 44 (29.7) | 43 (36.8) |  |  |
| Middle-West | 31 (6.1) |  | 46 (17.4) | 24 (16.2) | 22 (18.8) |  |  |
| Acute Leukemia |  |  |  |  |  |  |  |
| ALL | - |  | 169 (63.8) | 97 (65.5) | 72 (61.5) |  | - |
| AML | - |  | 96 (36.2) | 51 (34.5) | 45 (38.5) |  |  |
| *MLL* status |  |  |  |  |  |  |  |
| Rearranged | - |  | 121 (50.6) | 87 (61.7) | 34 (34.7) |  | - |
| Germline | - |  | 118 (49.4) | 54 (38.3) | 64 (65.3) |  |  |

^a^As defined by the mother of the index; ^b^Corresponds to the place of birth of the index; ^c^Refers to the comparison between controls and all cases.
