# Supplementary material for: ARID5B polymorphism confers an increased risk to acquire specific MLL rearrangements in early childhood leukemia
Source: BMC Cancer. 2014 Feb 25;14:127. doi: 10.1186/1471-2407-14-127 (PMC3948138; doi:10.1186/1471-2407-14-127)
Supplement: Additional file 2: Table S2 — The distribution of allele frequencies among controls and cases within the major acute leukemia subtypes, Brazil, 2003-2013. [file 1471-2407-14-127-S2.doc]

**Additional file 2: Table S2.** The distribution of allele frequencies among controls and cases within the major acute leukemia subtypes, Brazil, 2003-2013

|  | Controls |  | ALL | | | | | AML^c^ | |
| --- | --- | --- | --- | --- | --- | --- | --- | --- | --- |
|  |  |  | Pro-B ALL (CD10 neg) | |  | c-ALL (CD10 pos) | |  |  |
|  | n |  | n | OR (95% CI) ^a,b^ |  | n | OR (95% CI) ^a,b^ | n | OR (95% CI) ^a,b^ |
| *IKZF1* |  |  |  |  |  |  |  |  |  |
| rs11978267 |  |  |  |  |  |  |  |  |  |
| AA | 271 |  | 44 | 1.00 |  | 36 | 1.00 | 59 | 1.00 |
| AG | 182 |  | 29 | 1.23 (0.68-2.24) |  | 33 | 1.66 (0.93-2.97) | 28 | 0.80 (0.45-1.43) |
| GG | 37 |  | 4 | 0.64 (0.18-2.27) |  | 8 | 1.92 (0.70-5.26) | 6 | 0.83 (0.28-2.43) |
| AG+GG | 219 |  | 33 | 1.13 (0.64-2.00) |  | 41 | 1.71 (0.99-2.97) | 34 | 0.81 (0.47-1.39) |
| *ARID5B* |  |  |  |  |  |  |  |  |  |
| rs10821936 |  |  |  |  |  |  |  |  |  |
| TT | 200 |  | 23 | 1.00 |  | 19 | 1.00 | 28 | 1.00 |
| TC | 205 |  | 44 | **2.68 (1.37-5.23)** |  | 39 | **2.53 (1.30-4.94)** | 38 | 1.72 (0.93-3.19) |
| CC | 68 |  | 15 | **2.67 (1.12-6.36)** |  | 17 | **3.05 (1.35-6.93)** | 21 | **2.39 (1.10-5.17)** |
| TC+CC | 273 |  | 59 | **2.54 (1.36-4.70)** |  | 56 | **2.63 (1.41-4.90)** | 59 | **1.91 (1.08-3.37)** |
| rs10994982 |  |  |  |  |  |  |  |  |  |
| GG | 96 |  | 12 | 1.00 |  | 6 | 1.00 | 10 | 1.00 |
| GA | 214 |  | 40 | 1.57 (0.69-3.57) |  | 35 | **2.73 (1.03-7.29)** | 37 | 1.67 (0.72-3.86) |
| AA | 163 |  | 32 | 1.59 (0.69-3.68) |  | 36 | **3.70 (1.38-9.91)** | 38 | 2.13 (0.90-5.03) |
| GA+AA | 377 |  | 72 | 1.57 (0.73-3.36) |  | 71 | **3.13 (1.24-7.95)** | 75 | 1.85 (0.84-4.08) |
| *CEBPE* |  |  |  |  |  |  |  |  |  |
| rs2239633 |  |  |  |  |  |  |  |  |  |
| AA | 62 |  | 11 | 1.00 |  | 10 | 1.00 | 18 | 1.00 |
| AG | 201 |  | 41 | 1.43 (0.58-3.55) |  | 27 | 1.10 (0.45-2.68) | 43 | 0.94 (0.44-2.00) |
| GG | 220 |  | 28 | 0.66 (0.28-1.57) |  | 43 | 1.18 (0.52-2.71) | 30 | 0.46 (0.21-1.01) |
| AG+GG | 421 |  | 69 | 0.97 (0.43-2.17) |  | 70 | 1.13 (0.51-2.52) | 73 | 0.65 (0.33-1.31) |

ALL, acute lymphoblastic leukemia; AML, acute myeloid leukemia; CI, confidence intervals; n, number of individuals; neg, negative; OR, odds ratio; pos, positive; ^a^Adjusted on age; ^b^Adjusted on skin color; ^c^All FAB subtypes, except acute promyelocitic leukemia
