# Supplementary material for: ARID5B polymorphism confers an increased risk to acquire specific MLL rearrangements in early childhood leukemia
Source: BMC Cancer. 2014 Feb 25;14:127. doi: 10.1186/1471-2407-14-127 (PMC3948138; doi:10.1186/1471-2407-14-127)
Supplement: Additional file 3: Table S3 — The distribution of allele frequencies among controls and cases within the two major acute leukemia subtypes by skin colour and by MLL gene status, Brazil, 2003-2013. [file 1471-2407-14-127-S3.doc]

**Additional file 3: Table S3.** The distribution of allele frequencies among controls and cases within the two major acute leukemia subtypes by skin colour and by *MLL* gene status, Brazil, 2003-2013

|  |  | Controls | |  | Overall cases | | | | | | | | | | |
| --- | --- | --- | --- | --- | --- | --- | --- | --- | --- | --- | --- | --- | --- | --- | --- |
|  |  |  |  |  | White | | | | |  | Non-White | | | | |
|  |  | White | Non-White |  | *MLL*-germline (n=75) | |  | *MLL*-r (n=76) | |  | *MLL*-germline (n=43) | |  | *MLL*-r (n=45) | |
|  |  | n | n |  | n | OR (95% CI) |  | n | OR (95% CI) |  | n | OR (95% CI) |  | n | OR (95% CI) |
| *IKZF1* |  |  |  |  |  |  |  |  |  |  |  |  |  |  |  |
| rs11978267 |  |  |  |  |  |  |  |  |  |  |  |  |  |  |  |
| AA |  | 160 | 111 |  | 36 | 1.00 |  | 46 | 1.00 |  | 19 | 1.00 |  | 26 | 1.00 |
| AG |  | 110 | 72 |  | 27 | 1.09 (0.63-1.90) |  | 19 | 0.60 (0.33-1.08) |  | 23 | 1.76 (0.91-3.39) |  | 14 | 0.78 (0.39-1.57) |
| GG |  | 23 | 14 |  | 8 | 1.55 (0.64-3.73) |  | 5 | 0.76 (0.27-2.10) |  | 1 | 0.37 (0.05-2.87) |  | 1 | 0.27 (0.03-2.07) |
| AG+GG |  | 133 | 86 |  | 35 | 1.17 (0.70-1.97) |  | 24 | 0.63 (0.36-1.08) |  | 24 | 1.52 (0.80-2.89) |  | 15 | 0.69 (0.35-1.36) |
| *ARID5B* |  |  |  |  |  |  |  |  |  |  |  |  |  |  |  |
| rs10821936 |  |  |  |  |  |  |  |  |  |  |  |  |  |  |  |
| TT |  | 117 | 83 |  | 20 | 1.00 |  | 18 | 1.00 |  | 12 | 1.00 |  | 10 | 1.00 |
| TC |  | 129 | 76 |  | 30 | 1.36 (0.73-2.53) |  | 41 | **2.06 (1.12-3.79)** |  | 16 | 1.21 (0.55-2.66) |  | 26 | **2.36 (1.09-5.10)** |
| CC |  | 37 | 31 |  | 17 | **2.69 (1.28-5.66)** |  | 12 | 2.11 (0.93-4.78) |  | 14 | **3.69 (1.57-8.68)** |  | 6 | 1.90 (0.65-5.57) |
| TC+CC |  | 166 | 107 |  | 47 | 1.66 (0.93-2.94) |  | 53 | **2.08 (1.16-3.72)** |  | 30 | 1.76 (0.87-3.58) |  | 32 | **2.26 (1.07-4.77)** |
| rs10994982 |  |  |  |  |  |  |  |  |  |  |  |  |  |  |  |
| GG |  | 65 | 31 |  | 7 | 1.00 |  | 9 | 1.00 |  | 4 | 1.00 |  | 6 | 1.00 |
| GA |  | 125 | 89 |  | 35 | **2.60 (1.09-6.18)** |  | 35 | 2.02 (0.92-4.46) |  | 16 | 2.08 (0.67-6.48) |  | 16 | 1.39 (0.52-3.71) |
| AA |  | 96 | 67 |  | 25 | 2.42 (0.99-5.92) |  | 27 | 2.03 (0.90-4.60) |  | 21 | **3.55 (1.17-10.84)** |  | 22 | 2.48 (0.95-6.46) |
| GA+AA |  | 221 | 156 |  | 60 | **2.52 (1.10-5.78)** |  | 62 | 2.02 (0.96-4.30) |  | 37 | 2.72 (0.94-7.92) |  | 38 | 1.86 (0.75-4.60) |
| *CEBPE* |  |  |  |  |  |  |  |  |  |  |  |  |  |  |  |
| rs2239633 |  |  |  |  |  |  |  |  |  |  |  |  |  |  |  |
| AA |  | 39 | 23 |  | 9 | 1.00 |  | 11 | 1.00 |  | 11 | 1.00 |  | 4 | 1.00 |
| AG |  | 124 | 77 |  | 33 | 1.15 (0.51-2.62) |  | 37 | 1.06 (0.49-2.27) |  | 11 | **0.31 (0.13-1.78)** |  | 20 | 1.57 (0.51-4.88) |
| GG |  | 131 | 89 |  | 27 | 0.89 (0.39-2.06) |  | 24 | 0.65 (0.29-1.44) |  | 21 | 0.57 (0.25-1.28) |  | 20 | 1.49 (0.48-4.61) |
| AG+GG |  | 255 | 166 |  | 60 | 1.02 (0.47-2.22) |  | 61 | 0.85 (0.41-1.75) |  | 32 | **0.44 (0.21-0.95)** |  | 40 | 1.53 (0.52-4.51) |

ALL, acute lymphoblastic leukemia; AML, acute myeloid leukemia; n, number of individuals; OR, odds ratio; CI, confidence intervals; ^a^The age strata in AML is not relevant as it is for ALL, therefore, the AML cases have not been sub-divided by age

**Table 1.** continued

|  | Infant ALL (≤12 months) | | | | | | | | | | |
| --- | --- | --- | --- | --- | --- | --- | --- | --- | --- | --- | --- |
|  | White | | | | |  | Non-White | | | | |
|  | *MLL*-germline (n=19) | |  | *MLL*-r (n=46) | |  | *MLL*-germline (n=6) | |  | *MLL*-r (n=21) | |
|  | n | OR (95% CI) |  | n | OR (95% CI) |  | n | OR (95% CI) |  | n | OR (95% CI) |
|  |  |  |  |  |  |  |  |  |  |  |  |
|  |  |  |  |  |  |  |  |  |  |  |  |
|  | 5 | 1.00 |  | 26 | 1.00 |  | 3 | 1.00 |  | 11 | 1.00 |
|  | 7 | 2.04 (0.63-6.58) |  | 11 | 0.62 (0.29-1.30) |  | 3 | 1.54 (0.30-7.85) |  | 6 | 0.84 (0.30-2.37) |
|  | 4 | **5.57 (1.39-22.24)** |  | 3 | 0.80 (0.22-2.87) |  | 0 | - |  | 1 | 0.72 (0.09-6.01) |
|  | 11 | 2.65 (0.90-7.81) |  | 14 | 0.65 (0.33-1.29) |  | 3 | 1.29 (0.25-6.55) |  | 7 | 0.82 (0.31-2.21) |
|  |  |  |  |  |  |  |  |  |  |  |  |
|  |  |  |  |  |  |  |  |  |  |  |  |
|  | 4 | 1.00 |  | 12 | 1.00 |  | 3 | 1.00 |  | 4 | 1.00 |
|  | 9 | 2.04 (0.61-6.80) |  | 29 | **2.19 (1.07-4.49)** |  | 3 | 1.09 (0.21-5.58) |  | 14 | **3.82 (1.21-12.12)** |
|  | 4 | 3.16 (0.75-13.27) |  | 3 | 0.79 (0.21-2.95) |  | 0 | - |  | 3 | 2.01 (0.43-9.49) |
|  | 13 | 2.29 (0.73-7.20) |  | 32 | 1.88 (0.93-3.80) |  | 3 | 0.78 (0.15-3.94) |  | 17 | **3.30 (1.07-10.17)** |
|  |  |  |  |  |  |  |  |  |  |  |  |
|  | 2 | 1.00 |  | 5 | 1.00 |  | 1 | 1.00 |  | 4 | 1.00 |
|  | 10 | 2.60 (0.55-12.22) |  | 24 | 2.50 (0.91-6.85) |  | 3 | 1.04 (0.10-10.42) |  | 8 | 0.70 (0.20-2.48) |
|  | 9 | 3.05 (0.64-14.56) |  | 15 | 2.03 (0.70-5.86) |  | 3 | 1.39 (0.14-13.89) |  | 9 | 1.04 (0.30-3.64) |
|  | 19 | 2.79 (0.63-12.31) |  | 39 | 2.29 (0.87-6.06) |  | 6 | 1.19 (0.14-10.25) |  | 17 | 0.84 (0.27-2.68) |
|  |  |  |  |  |  |  |  |  |  |  |  |
|  |  |  |  |  |  |  |  |  |  |  |  |
|  | 3 | 1.00 |  | 8 | 1.00 |  | 2 | 1.00 |  | 1 | 1.00 |
|  | 4 | 0.42 (0.09-1.96) |  | 26 | 1.02 (0.43-2.44) |  | 1 | 0.15 (0.01-1.72) |  | 11 | 3.29 (0.40-26.82) |
|  | 9 | 0.89 (0.23-3.46) |  | 8 | **0.30 (0.10-0.84)** |  | 3 | 0.39 (0.06-2.46) |  | 8 | 2.07 (0.25-17.38) |
|  | 13 | 0.66 (0.18-2.43) |  | 34 | 0.65 (0.28-1.51) |  | 4 | 0.28 (0.05-1.60) |  | 19 | 2.63 (0.34-20.61) |

**Table 1.** continued

|  | ALL (13-24 months) | | | | | | | | | | |
| --- | --- | --- | --- | --- | --- | --- | --- | --- | --- | --- | --- |
|  | White | | | | |  | Non-White | | | | |
|  | *MLL*-germline (n=23) | |  | *MLL*-r (n=11) | |  | *MLL*-germline (n=18) | |  | *MLL*-r (n=9) | |
|  | n | OR (95% CI) |  | n | OR (95% CI) |  | n | OR (95% CI) |  | n | OR (95% CI) |
|  |  |  |  |  |  |  |  |  |  |  |  |
|  |  |  |  |  |  |  |  |  |  |  |  |
|  | 7 | 1.00 |  | 6 | 1.00 |  | 7 | 1.00 |  | 7 | 1.00 |
|  | 12 | 2.49 (0.95-6.53) |  | 5 | 1.21 (0.36-4.07) |  | 11 | 2.42 (0.90-6.54) |  | 2 | 0.44 (0.09-2.18) |
|  | 3 | 2.98 (0.72-12.35) |  | 0 | - |  | 0 | - |  | 0 | - |
|  | 15 | **2.58 (1.02-6.51)** |  | 5 | 1.00 (0.30-3.36) |  | 11 | 2.03 (0.75-5.45) |  | 2 | 0.37 (0.07-1.82) |
|  |  |  |  |  |  |  |  |  |  |  |  |
|  |  |  |  |  |  |  |  |  |  |  |  |
|  | 4 | 1.00 |  | 2 | 1.00 |  | 4 | 1.00 |  | 2 | 1.00 |
|  | 7 | 1.59 (0.45-5.56) |  | 3 | 1.36 (0.22-8.29) |  | 8 | 2.18 (0.63-7.55) |  | 6 | 3.28 (0.64-16.73) |
|  | 9 | **7.11 (2.07-24.45)** |  | 5 | **7.91 (1.47-42.46)** |  | 5 | 3.35 (0.84-13.28) |  | 1 | 1.34 (0.12-15.29) |
|  | 16 | 2.82 (0.92-8.64) |  | 8 | 2.82 (0.59-13.52) |  | 13 | 2.50 (0.79-8.02) |  | 8 | 3.10 (0.64-15.00) |
|  |  |  |  |  |  |  |  |  |  |  |  |
|  | 1 | 1.00 |  | 1 | 1.00 |  | 2 | 1.00 |  | 2 | 1.00 |
|  | 8 | 4.16 (0.51-33.98) |  | 5 | 2.60 (0.30-22.72) |  | 8 | 1.39 (0.28-6.92) |  | 3 | 0.52 (0.08-3.27) |
|  | 10 | 6.77 (0.85-54.18) |  | 4 | 2.71 (0.30-24.78) |  | 8 | 1.85 (0.37-9.23) |  | 4 | 0.93 (0.16-5.33) |
|  | 18 | 5.29 (0.69-40.42) |  | 9 | 2.65 (0.33-21.28) |  | 16 | 1.59 (0.35-7.27) |  | 7 | 0.70 (0.14-3.51) |
|  |  |  |  |  |  |  |  |  |  |  |  |
|  |  |  |  |  |  |  |  |  |  |  |  |
|  | 3 | 1.00 |  | 1 | 1.00 |  | 2 | 1.00 |  | 1 | 1.00 |
|  | 9 | 0.94 (0.24-3.66) |  | 3 | 0.94 (0.10-9.33) |  | 4 | 0.60 (0.10-3.47) |  | 2 | 0.60 (0.05-6.89) |
|  | 11 | 1.09 (0.29-4.11) |  | 7 | 2.08 (0.25-17.46) |  | 12 | 1.55 (0.32-7.42) |  | 6 | 1.55 (0.18-13.53) |
|  | 20 | 1.02 (0.29-3.59) |  | 11 | 1.68 (0.21-13.40) |  | 16 | 1.11 (0.24-5.14) |  | 8 | 1.11 (0.13-9.27) |

**Table 1.** continued

|  | AML | | | | | | | | | | |
| --- | --- | --- | --- | --- | --- | --- | --- | --- | --- | --- | --- |
|  | White | | | | |  | Non-White | | | | |
|  | *MLL*-germline (n=33) | |  | *MLL*-r (n=19) | |  | *MLL*-germline (n=19) | |  | *MLL*-r (n=15) | |
|  | n | OR (95% CI) |  | n | OR (95% CI) |  | n | OR (95% CI) |  | n | OR (95% CI) |
|  |  |  |  |  |  |  |  |  |  |  |  |
|  |  |  |  |  |  |  |  |  |  |  |  |
|  | 24 | 1.00 |  | 14 | 1.00 |  | 9 | 1.00 |  | 8 | 1.00 |
|  | 8 | 0.48 (0.21-1.12) |  | 3 | 0.31 (0.09-1.11) |  | 9 | 1.54 (0.58-4.07) |  | 6 | 1.16 (0.39-3.47) |
|  | 1 | 0.29 (0.04-2.25) |  | 2 | 0.99 (0.21-4.66) |  | 1 | 0.88 (0.10-7.48) |  | 0 | - |
|  | 9 | 0.45 (0.20-1.00) |  | 5 | 0.43 (0.15-1.22) |  | 10 | 1.43 (0.56-3.68) |  | 6 | 0.97 (0.32-2.89) |
|  |  |  |  |  |  |  |  |  |  |  |  |
|  |  |  |  |  |  |  |  |  |  |  |  |
|  | 12 | 1.00 |  | 4 | 1.00 |  | 4 | 1.00 |  | 5 | 1.00 |
|  | 14 | 1.06 (0.47-2.38) |  | 9 | 2.04 (0.61-6.80) |  | 6 | 1.64 (0.45-6.03) |  | 5 | 1.09 (0.30-3.92) |
|  | 4 | 1.05 (0.32-3.47) |  | 4 | 3.16 (0.75-13.27) |  | 3 | 2.01 (0.43-9.49) |  | 9 | **4.82 (1.50-15.50)** |
|  | 18 | 1.06 (0.49-2.28) |  | 13 | 2.29 (0.73-7.20) |  | 9 | 1.75 (0.52-5.87) |  | 14 | 2.17 (0.75-6.27) |
|  |  |  |  |  |  |  |  |  |  |  |  |
|  | 4 | 1.00 |  | 3 | 1.00 |  | 2 | 1.00 |  | 1 | 1.00 |
|  | 17 | 2.21 (0.71-6.84) |  | 6 | 1.04 (0.25-4.29) |  | 5 | 0.87 (0.16-4.72) |  | 5 | 1.74 (0.20-15.49) |
|  | 8 | 1.35 (0.39-4.68) |  | 8 | 1.81 (0.46-7.06) |  | 10 | 2.31 (0.48-11.20) |  | 9 | 4.16 (0.51-34.33) |
|  | 25 | 1.84 (0.62-5.47) |  | 14 | 1.37 (0.38-4.92) |  | 15 | 1.49 (0.32-6.85) |  | 14 | 2.78 (0.35-21.94) |
|  |  |  |  |  |  |  |  |  |  |  |  |
|  |  |  |  |  |  |  |  |  |  |  |  |
|  | 3 | 1.00 |  | 3 | 1.00 |  | 7 | 1.00 |  | 3 | 1.00 |
|  | 20 | 2.10 (0.59-7.43) |  | 8 | 0.84 (0.21-3.32) |  | 6 | **0.26 (0.08-0.84)** |  | 7 | 0.70 (0.17-2.91) |
|  | 7 | 0.69 (0.17-2.81) |  | 8 | 0.79 (0.20-3.14) |  | 6 | **0.22 (0.07-0.72)** |  | 5 | 0.43 (0.10-1.94) |
|  | 27 | 1.38 (0.40-4.75) |  | 16 | 0.82 (0.23-2.93) |  | 12 | **0.24 (0.08-0.66)** |  | 12 | 0.55 (0.15-2.11) |
