# Supplementary material for: ARID5B polymorphism confers an increased risk to acquire specific MLL rearrangements in early childhood leukemia
Source: BMC Cancer. 2014 Feb 25;14:127. doi: 10.1186/1471-2407-14-127 (PMC3948138; doi:10.1186/1471-2407-14-127)
Supplement: Additional file 4: Table S4. — The risk associations between genetic variants and MLL status in overall and specific subtypes of acute leukemia, Brazil, 2003-2013. [file 1471-2407-14-127-S4.doc]

**Additional file 4: Table S4:** The risk associations between genetic variants and *MLL* status in overall and specific subtypes of acute leukemia**,** Brazil, 2003-2013

|  |  | Controls |  | Overall cases | | | | |  | ALL | | | | |  | AML | | | | |
| --- | --- | --- | --- | --- | --- | --- | --- | --- | --- | --- | --- | --- | --- | --- | --- | --- | --- | --- | --- | --- |
|  |  |  |  | *MLL*-germline (n=118) | |  | *MLL*-r (n=121) | |  | *MLL*-germline (n=62) | |  | *MLL*-r (n=87) | |  | *MLL*-germline (n=52) | |  | *MLL*-r (n=34) | |
|  |  | N |  | n | OR (95% CI)^a,b^ |  | n | OR (95% CI)^a,b^ |  | n | OR (95% CI)^a,b^ |  | n | OR (95% CI)^a,b^ |  | n | OR (95% CI)^b^ |  | n | OR (95% CI)^b^ |
| *IKZF1* |  |  |  |  |  |  |  |  |  |  |  |  |  |  |  |  |  |  |  |  |
| rs11978267 |  |  |  |  |  |  |  |  |  |  |  |  |  |  |  |  |  |  |  |  |
| AA |  | 271 |  | 55 | 1.00 |  | 72 | 1.00 |  | 22 | 1.00 |  | 50 | 1.00 |  | 33 | 1.00 |  | 22 | 1.00 |
| AG |  | 182 |  | 50 | 1.63 (0.97-2.72) |  | 33 | 0.79 (0.46-1.38) |  | 33 | **2.73 (1.44-5.18)** |  | 24 | 0.85 (0.46-1.57) |  | 17 | 0.89 (0.45-1.77) |  | 9 | 0.67 (0.28-1.59) |
| GG |  | 37 |  | 9 | 1.41 (0.54-3.70) |  | 6 | 0.60 (0.20-1.79) |  | 7 | 2.91 (0.98-8.63) |  | 4 | 0.55 (0.16-1.91) |  | 2 | 0.49 (0.10-2.38) |  | 2 | 0.73 (0.15-3.62) |
| AG+GG |  | 219 |  | 59 | 1.60 (0.98-2.61) |  | 39 | 0.76 (0.45-1.28) |  | 40 | **2.77 (1.50-5.10)** |  | 28 | 0.80 (0.45-1.43) |  | 19 | 0.82 (0.42-1.58) |  | 11 | 0.69 (0.31-1.54) |
| *ARID5B* |  |  |  |  |  |  |  |  |  |  |  |  |  |  |  |  |  |  |  |  |
| rs10821936 |  |  |  |  |  |  |  |  |  |  |  |  |  |  |  |  |  |  |  |  |
| TT |  | 200 |  | 32 | 1.00 |  | 28 | 1.00 |  | 15 | 1.00 |  | 20 | 1.00 |  | 17 | 1.00 |  | 8 | 1.00 |
| TC |  | 205 |  | 46 | **1.84 (1.02-3.30)** |  | 67 | **3.14 (1.72-5.74)** |  | 27 | **2.35 (1.11-4.95)** |  | 50 | **3.55 (1.80-6.99)** |  | 19 | 1.38 (0.65-2.95) |  | 15 | 2.52 (0.97-6.57) |
| CC |  | 68 |  | 31 | **3.39 (1.66-6.91)** |  | 18 | **2.36 (1.06-5.24)** |  | 18 | **4.22 (1.80-9.90)** |  | 17 | 2.10 (0.83-5.27) |  | 13 | 2.43 (0.98-6.04) |  | 7 | 2.85 (0.90-8.97) |
| TC+CC |  | 273 |  | 77 | **2.22 (1.30-3.80)** |  | 88 | **2.87 (1.63-5.05)** |  | 45 | **2.77 (1.40-5.47)** |  | 67 | **3.04 (1.61-5.72)** |  | 32 | 1.70 (0.86-3.37) |  | 22 | **2.56 (1.05-6.23)** |
| rs10994982 |  |  |  |  |  |  |  |  |  |  |  |  |  |  |  |  |  |  |  |  |
| GG |  | 96 |  | 11 | 1.00 |  | 15 | 1.00 |  | 28 | 1.00 |  | 12 | 1.00 |  | 6 | 1.00 |  | 3 | 1.00 |
| GA |  | 214 |  | 51 | 2.13 (0.97-4.70) |  | 51 | 1.53 (0.71-5.27) |  | 29 | 2.62 (0.91-7.53) |  | 40 | 1.50 (0.66-3.41) |  | 22 | 1.69 (0.62-4.64) |  | 11 | 1.67 (0.42-6.66) |
| AA |  | 163 |  | 46 | **2.46 (1.09-5.57)** |  | 49 | 1.92 (0.89-4.13) |  | 5 | **3.45 (1.19-10.04)** |  | 32 | 1.51 (0.65-3.50) |  | 18 | 1.58 (0.54-4.60) |  | 17 | 3.28 (0.86-12.51) |
| GA+AA |  | 377 |  | 97 | **2.29 (1.08-4.85)** |  | 100 | 1.70 (0.84-3.43) |  | 34 | **2.97 (1.08-8.12)** |  | 72 | 1.50 (0.70-3.22) |  | 40 | 1.69 (0.65-4.41) |  | 28 | 2.26 (0.63-8.14) |
| *CEBPE* |  |  |  |  |  |  |  |  |  |  |  |  |  |  |  |  |  |  |  |  |
| rs2239633 |  |  |  |  |  |  |  |  |  |  |  |  |  |  |  |  |  |  |  |  |
| AA |  | 62 |  | 20 | 1.00 |  | 15 | 1.00 |  | 10 | 1.00 |  | 9 | 1.00 |  | 10 | 1.00 |  | 6 | 1.00 |
| AG |  | 201 |  | 44 | 0.86 (0.41-1.79) |  | 57 | 1.43 (0.63-3.23) |  | 18 | 0.71 (0.28-1.78) |  | 42 | 2.08 (0.77-5.57) |  | 26 | 1.01 (0.41-2.47) |  | 15 | 0.94 (0.32-2.77) |
| GG |  | 220 |  | 48 | 0.69 (0.34-1.41) |  | 44 | 0.78 (0.36-1.67) |  | 35 | 0.96 (0.41-2.26) |  | 31 | 0.91 (0.37-2.23) |  | 13 | **0.36 (0.13-0.99)** |  | 13 | 0.57 (0.19-1.70) |
| AG+GG |  | 421 |  | 92 | 0.76 (0.39-1.47) |  | 101 | 1.04 (0.50-2.15) |  | 53 | 0.86 (0.38-1.93) |  | 73 | 1.29 (0.55-3.03) |  | 39 | 0.62 (0.27-1.44) |  | 28 | 0.72 (0.26-1.96) |

*MLL*-GL, *MLL* germline; *MLL*-r, *MLL* rearranged; ALL, acute lymphoblastic leukemia; AML, acute myeloid leukemia; n, number of individuals; OR, odds ratio; CI, confidence intervals; ^a^Adjusted on age; ^b^Adjusted on skin color.
