# Supplementary material for: ARID5B polymorphism confers an increased risk to acquire specific MLL rearrangements in early childhood leukemia
Source: BMC Cancer. 2014 Feb 25;14:127. doi: 10.1186/1471-2407-14-127 (PMC3948138; doi:10.1186/1471-2407-14-127)
Supplement: Additional file 5: Table S5 — Cumulative risk effects of IKZF1, ARID5B and CEBPE genetic variants according to leukemia subtype and MLL status, Brazil, 2003-2013. [file 1471-2407-14-127-S5.doc]

***Additional file 5: Table S5:*** *Cumulative risk effects of IKZF1, ARID5B and CEBPE genetic variants according to leukemia subtype and MLL status, Brazil, 2003-2013*

|  | Controls |  | Overall cases | |  | ALL | | | | |  | AML | |  | *MLL* germline | |  | *MLL*-r | |
| --- | --- | --- | --- | --- | --- | --- | --- | --- | --- | --- | --- | --- | --- | --- | --- | --- | --- | --- | --- |
|  |  |  |  |  |  | ≤12 months | |  | 13-24 months | |  |  |  |  |  |  |  |  |  |
|  | n |  | n | OR (95% CI)^b^ |  | n | OR (95% CI)^b^ |  | n | OR (95% CI)^b^ |  | n | OR (95% CI)^b^ |  | n | OR (95% CI)^b^ |  | n | OR (95% CI)^b^ |
| Number of risk alleles^a^ |  |  |  |  |  |  |  |  |  |  |  |  |  |  |  |  |  |  |  |
| 0-1 | 41 |  | 9 | 1.00 |  | 4 | 1.00 |  | 0 | - |  | 5 | 1.00 |  | 4 | 1.00 |  | 4 | 1.00 |
| 2 | 68 |  | 27 | 1.94 (0.92-4.12) |  | 13 | 2.06 (0.71-5.98) |  | 5 | 1.00 |  | 9 | 1.09 (0.34-3.347) |  | 10 | 1.50 (0.44-5.12) |  | 14 | 2.13 (0.66-6.96) |
| 3 | 123 |  | 53 | 1.43 (0.98-2.06) |  | 23 | 1.41 (0.83-2.39) |  | 10 | 1.06 (0.61-1.86) |  | 20 | 1.15 (0.68-1.95) |  | 23 | 1.38 (0.79-2.42) |  | 27 | 1.50 (0.86-2.62) |
| 4 | 101 |  | 52 | **1.34 (1.04-1.73)** |  | 15 | 1.17 (0.81-1.70) |  | 17 | 1.32 (0.93-1.87) |  | 20 | 1.17 (0.82-1.66) |  | 23 | 1.33 (0.91-1.93) |  | 24 | 1.35 (0.93-1.95) |
| 5 | 77 |  | 35 | 1.21 (0.99-1.48) |  | 15 | 1.19 (0.90-1.59) |  | 11 | 1.21 (0.91-1.60) |  | 9 | 0.99 (0.74-1.33) |  | 13 | 1.16 (0.86-1.56) |  | 19 | 1.26 (0.95-1.68) |
| 6-8 | 59 |  | 43 | **1.27 (1.09-1.48)** |  | 11 | 1.17 (0.93-1.47) |  | 19 | **1.34 (1.09-1.66)** |  | 13 | 1.12 (0.89-1.39) |  | 24 | **1.33 (1.06-1.67)** |  | 17 | 1.25 (0.99-1.58) |

ALL, acute lymphoblastic leukemia; AML, acute myeloid leukemia; n, number of individuals; OR, odds ratio; CI, confidence intervals; ^a^Refers to the cumulative number of variant alleles of *IKZF1*, *ARID5B* and *CEBPE* genes in the same patient; only patients analyzed for all four loci have been included in this analyses; ^b^Adjusted on skin color.
